# Comparison of blue-green solutions for urban flood mitigation: A multi-city large-scale analysis

**DOI:** 10.1371/journal.pone.0246429

**Published:** 2021-01-29

**Authors:** Elena Cristiano, Stefano Farris, Roberto Deidda, Francesco Viola

**Affiliations:** Dipartimento di Ingegneria civile, ambientale e architettura—Sezione Idraulica, Facoltà di Ingegneria Università degli Studi di Cagliari, Cagliari (CA), Italy; University of Parma, ITALY

## Abstract

Flooding risk in cities has been recently exacerbated by increased urbanization and climate change, often with catastrophic consequences in terms of casualties and economic losses. Rainwater harvesting systems and green roofs are recognized as being among the most effective blue-green mitigation measures. However, performances of these systems have currently been investigated only at laboratory or very-small local scales. In this work, we assess the potential benefit of the extensive installation of these solutions on all the rooftops of 9 cities, with different climatological and geographical characteristics. Both surface discharge reduction and delay between rainfall and runoff peak generation have been investigated. Green roofs ensure a larger average lag time between rainfall and runoff peaks than rainwater harvesting systems, without significant differences between intensive and extensive structures. On the other hand, the cost-efficiency analysis, considering the entire urban area, shows a higher retention capacity with a lower financial investment for rainwater harvesting rather than for green roofs in most cases. For extreme rainfall events, large-scale installation of rainwater harvesting systems coupled with intensive green roofs over the entire city have shown to be the most efficient solution, with a total discharge reduction that can vary from 5% to 15%, depending on the city characteristics and local climate.

## Introduction

There is a consensus among scientists that climate change is leading to an intensification of short-duration rainfall events, alternated with long dry periods [1–3]. At the same time, cities have become more and more urbanized, with a growth of urban density and impermeable surfaces [4–6]. The combination of these two phenomena makes our cities more prone to flood risk. To cope with excess rainwater, massive traditional engineering infrastructures (e.g. pipes and storage reservoirs) were built. However, traditional structures are costly and usually not flexible to adapt to future climate changes and urban development scenarios. Several nature-based solutions, e.g. green roofs, rainwater parks or permeable pavements, have been lately proposed and preferred to mitigate flood risk connected to extreme rainfall events at local scale [7–9]. Among the available traditional and nature-based measures, rainwater harvesting (RWH) systems and green roofs (GRs) [10, 11] are the most popular and efficient blue-green solutions for collecting and storing water from the roofs to reduce and delay flood peaks, and for these reasons they have been chosen for this study.

GRs are sustainable tools [12] that enable a portion of the rainfall volume to be stored in the soil layer, which is later absorbed by the vegetation roots and returned to the atmosphere by evapotranspiration [13–17]. The retained rainfall volume depends on the dimension of the roof, on the soil type and thickness, and on vegetation species. GRs present multiple benefits: besides flood mitigation, they guarantee biodiversity, help to lower the building and surrounding temperature [18], contribute to reducing pollution retaining contaminants in the soil and add aesthetic values to urban environments [19–21]. Moreover, during the current COVID-19 pandemic crisis, green infrastructures have largely shown to have a positive impact on human wellbeing and life quality [22, 23], suggesting that their installation will be largely considered in the near future [24]. GRs are generally classified as “extensive” when the soil thickness is less than 15 cm and as “intensive” when it is more, allowing a deeper space for the vegetation root development. A drawback of this tool is that GRs should be installed only on flat or semi-flat roofs, since installation on sloped surfaces requires additional structural elements and leads to a lower retention performance [16, 25–27].

Conversely, RWH systems can be installed regardless of the roof slope, since they collect rainfall from the rooftops and store it in water tanks generally located at ground level [28–30]. Although RWH systems have been developed to collect water in rural areas for irrigation [31], they can be easily adapted and installed in an urban context, with the aim to mitigate rainfall extremes [30]. Collected water, if properly treated and stored, can be reused for different purposes, such as irrigation or other non-drinking domestic uses, being a good support to the water supply system [32]. RWH, however, requires the availability of a large space to locate the water tank, posing some constraints in the general urban planning of the city.

The two aforementioned solutions have been generally studied at local point scale, focusing on the impact induced by a single building installation. However, it is fundamental to evaluate the mitigation capacity of these tools over an entire city or large neighbourhood in order to identify the most suitable solution, depending on the study area characteristics. Only a few works have investigated the effects of potential installation at medium city-scale of either GRs [33, 34], or RWH [35] systems, and none of them have considered the impact of combined solutions. In this work, we analyse the mitigation efficiency in terms of runoff generation reduction and runoff peak delay, achievable thanks to a large-scale installation of GRs and RWH systems on the entire urban areas. Through a cost-efficiency analysis, the outflow reduction is evaluated for different scenarios, which consider the installation of two different flood mitigation measures, i.e. GRs and RWH systems, separately and combined, focusing on the mitigation performance during extreme rainfall events.

Nine cities around the globe with different geomorphological characteristics and climate conditions have been selected to investigate the effects of changes in roof runoff contribution at urban scale: Vancouver, Airdrie, Waterloo, Montreal (Canada), Port au Prince (Haiti), London (United Kingdom), Cagliari (Italy), Wellington and Auckland (New Zealand). For the sake of generality of our results, we selected cities representing different climatological areas, including Oceanic, Mediterranean, Continental and Equatorial climates.

This paper is structured as follows. In the Methodology section we illustrate how to identify the average roof slope and to estimate the runoff reduction achievable with the installation of blue-green solutions. Results are presented and discussed in the following section, mainly focusing on the average roof slope distribution, on the lag time between rainfall and runoff and on the cost-efficiency analysis for extreme rainfall events. A conclusive paragraph summarizes the main findings and highlights the possible implications of this work for the development of smart and flood resilient cities and the future research steps.

## Methodology

**Fig 1:**
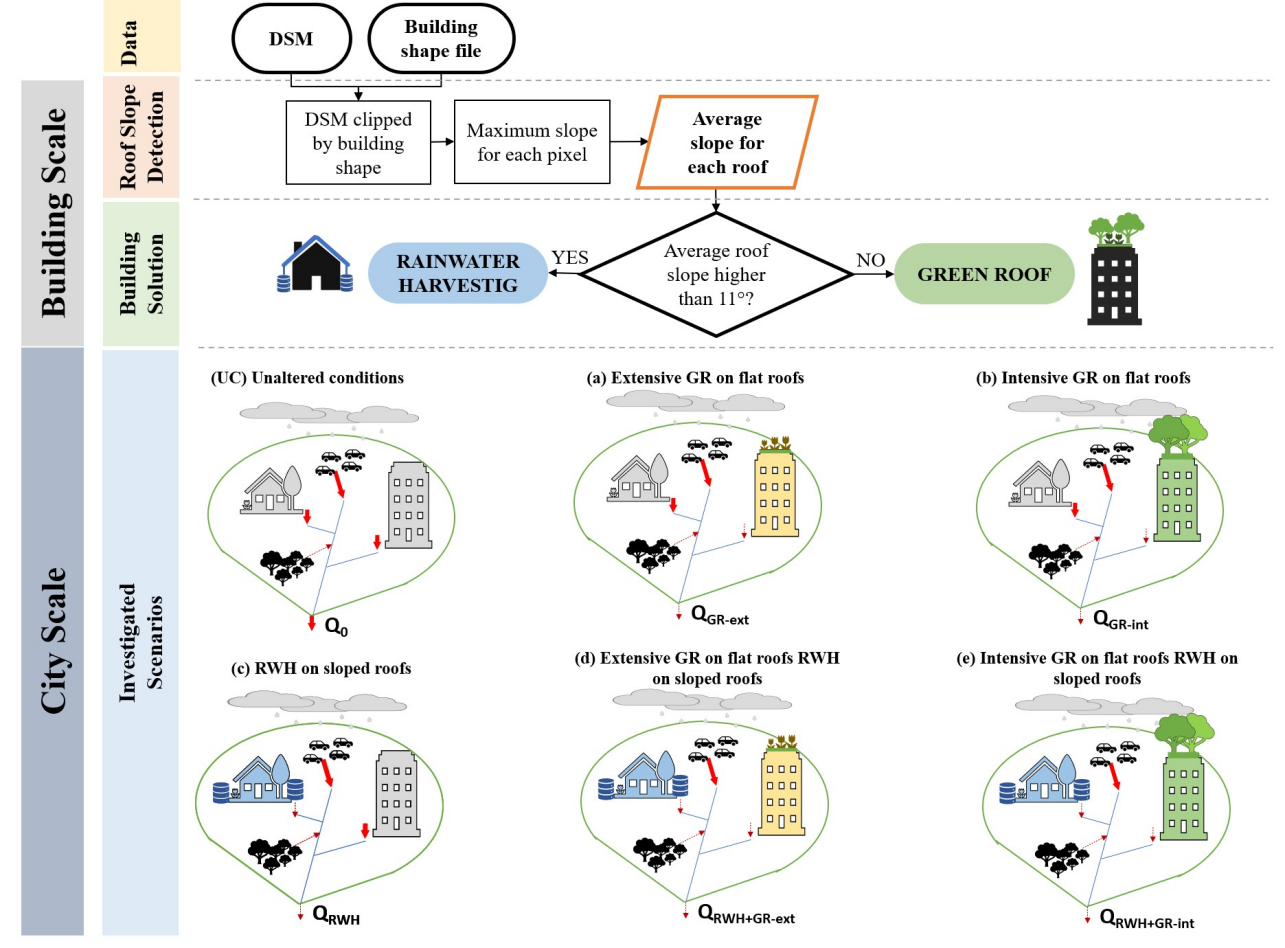
Schematic representation of the methodology used in this work to select the mitigation strategy to implement (GR or RWH, as a function of roof slope) and the analysed scenarios. The selection of the mitigation solution to install is done at “Building scale”, i.e. evaluating each building separately. The analysis of the discharge reduction for the different scenarios is developed at “City scale”, i.e. investigating the effects for an extended urban area, which can be a city or a large neighbourhood.

The methodology followed in this work to identify roof slope and to choose the most suitable blue-green solution, or combination of solutions, is summarized in Fig 1, which includes also the investigated scenarios. Since GRs have shown a higher retention capacity when installed on flat surfaces [25–27], they are assumed to be installed only on flat roofs, with an average slope less than 11° [36], while RWH systems are hypothesized for sloped surfaces

Fig 1(UC) illustrates a stylized urban area in unaltered conditions, in which the runoff *Q*_0_ comes from rooftops, roads, parking areas and green areas. Conversely, the other scenarios (Fig 1A–1E) schematically represent a green-blue city with GRs on flat roofs and/or RWH tanks under sloped ones to manage a reduced runoff Q*, which is then compared to the unaltered conditions:

- installation of extensive GRs over all the flat roofs in the city (*Q*_*GR−ext*_),
- installation of intensive GRs over all the flat roofs in the city (*Q*_*GR−int*_),
- installation of RWH tanks for all the sloped roofs in the city (*Q*_*RWH*_),
- installation of extensive GRs and RWH on flat and sloped roofs respectively (*Q*_*RWH*+*GR*−*ext*_),
- installation of intensive GRs and RWH on flat and sloped roofs respectively (*Q*_*RWH*+*GR*−*ext*_).

With the aim to understand the potential mitigation capacity of GRs and RWH systems under critical conditions, i.e. during extreme rainfall events, we evaluated, for the scenarios a, b and c, the flood discharge reduction considering an one-day event, with rainfall intensity equal to the 95%-quantile of the cumulative probability distribution of local daily rainfall time series (data sources available in the S1 Table). Subsequently, a long time series analysis with a related cost-efficiency analysis has been carried out for all the five scenarios proposed in Fig 1A–1E.

### Roof slope detection

Average rooftop slope, required in order to choose the best blue-green solution to apply, has been obtained by the combination of the Digital Surface Model (DSM) and building shape layer, available for many cities and commonly used for urban planning. The building shape layer was used as a mask to select only the DSM pixels belonging to the roof. For each pixel, the maximum slope was estimated and then averaged over the roof surface. In order to avoid boundary errors, slopes greater than 45° were neglected. DSMs with a resolution higher or equal to 2 m were analysed, with the aim to obtain accurate results. A coarser resolution would, in fact, increase the level of uncertainty of the obtained results.

**Fig 2:**
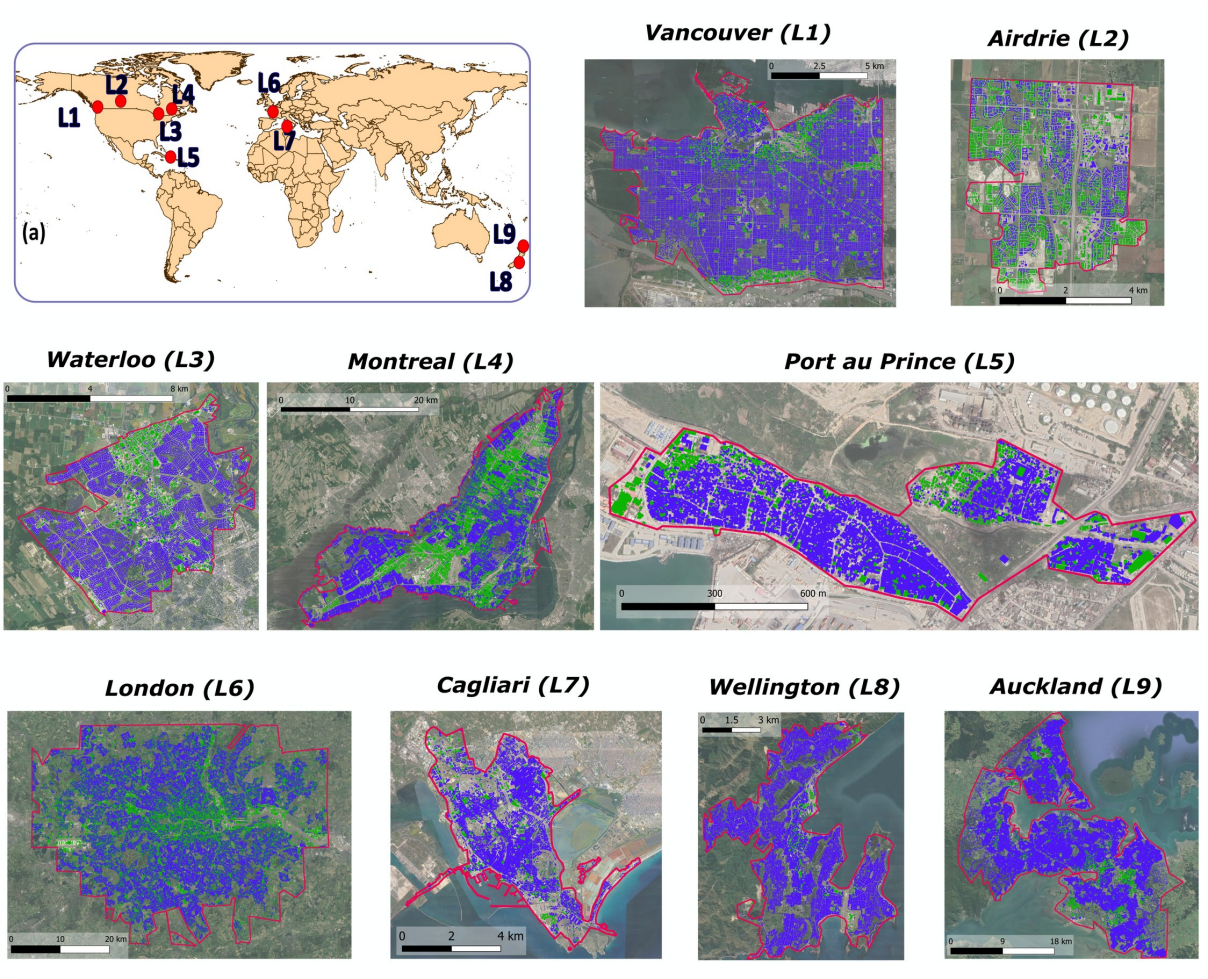
Geographical location of the 9 selected case studies. (a) Location of the 9 cities on a world map. (L1-L9) Boundaries of the study cases with investigated rooftops. Flat roofs are highlighted in green, while sloped roofs are represented in blue. Cities are ordered based on their geographic location from west to east. Maps have been developed with the help of QGIS 3.4 [37], using layers downloaded from the different geoportals listed in the S1 Table and satellite images derived from the Landsat website (http://landsat.visibleearth.nasa.gov/).

City boundaries have been defined, depending on the data availability, in order to highlight the densely populated areas, where the vulnerability to floods is higher. For most locations, analysis focuses only on the city centre. Selected city boundaries are shown in Fig 2L1–2L9.

### Outflow estimation

To estimate the total outflow *Q*_0_ from an urban catchment in unaltered conditions, the rational method [38] was applied. The discharge is defined as the product of rainfall rate *R*, the total area *A* and a coefficient *φ*, which depends on the imperviousness degree of the catchment:

$$Q_{0}=A\varphi R.$$

The coefficient *φ* is defined as weighted average over the area, considering the different types of surface (green areas, roofs and roads). The *φ* coefficient is assumed to be equal to 0.2 in green areas (e.g. urban parks, cemeteries), while for roofs it is set at 1 and 0.9 for streets, since private gardens are included in this last category.

The potential discharge reduction Δ*Q* for each scenario is defined as the normalized difference between *Q*_0_ and the mitigated discharge:

$$\Delta Q=\frac{Q_{0}-Q^{*}}{Q_{0}},$$

Where Q* is the mitigated discharge from green roofs (*Q*_*GR*_), rainwater harvesting tanks (*Q*_*RWH*_ and coupled systems, as described in the following paragraphs. The potential discharge reduction Δ*Q* is a dimensionless coefficient that globally quantifies the performance of the flood mitigation measures.

The GR retention capacity is estimated using the conceptual Ecohydrological Streamflow Model (EHSM) proposed by Viola, Pumo [39]. For each location, long historical rainfall time series and potential evapotranspiration series estimated by local temperature time series were used as inputs for the model.

EHSM is a parsimonious conceptual lumped model, based on water balance, developed to simulate the daily streamflow in semi-arid areas, which can be properly calibrated to represent the hydrological behaviour of GRs [40, 41]. The EHSM simulates the behaviour of a soil bucket and two parallel linear reservoirs, requiring rainfall and potential evapotranspiration rates at daily scale as numerical input. Four EHSM parameters need to be properly chosen to represent the GR behaviour. To describe the soil and vegetation characteristics, the model uses the active soil depth (*nZ*_*r*_), which is the product of soil depth (*Z*_*r*_) and porosity (*n*), the soil moisture values triggering the leakage (*S*_*fc*_), the hygroscopic point (*S*_*u*_) and the crop coefficient (*K*_*c*_). The soil used is assumed to be loamy sand, which is one of the most common soil type used for GRs, and the parameters *n*, *S*_*fc*_ and *S*_*u*_ are consequently derived from Laio, Porporato [42], which summarized the values of these parameters for several soil types. The thickness of the soil layer (*Z*_*r*_) is selected equal to 15 cm for the extensive configuration and 30 cm for the intensive one. The crop coefficient *K*_*c*_ represent the vegetation type and stress conditions and it is necessary to transform the potential evapotranspiration in evapotranspiration [43]. For grass in standard conditions the crop coefficient can be assumed equal to 1 [43]. EHSM enables to evaluate the retention capacity of GRs with different vegetation layer, changing the crop coefficient: vegetation characterized by a crassulacean acid metabolism, for example, are well represented by lower crop coefficient, with value close to 0.5 [44]. In this work, we assumed to install grass (*K*_*c*_ = 1) as top layer, which is the most flexible vegetation type for all the investigated locations.

Thanks to the simulation of the soil moisture dynamics, the EHSM enables the antecedent soil moisture conditions to be taken into consideration in the estimation of outflow from the GR.

The total outflow *Q*_*GR*_ is defined as the difference between the discharge *Q*_0_ for unaltered conditions and the daily volume retained by the GR, namely *q*_*GR*_, estimated with the ecohydrological model:

$$Q_{GR}=Q_{0}-q_{GR}$$

The discharge reduction due to the RWH is evaluated assuming the installation of a tank for each building. The maximum tank volume, *V*_*tank*_, was chosen based on the mitigation capacity we wanted to achieve. In this case, we assumed that the RWH tanks have a storing capacity equal to the volume of water conveyed by the rooftop during a daily extreme event. Specifically, we consider a daily rainfall rate equal to the 95%-quantile of the cumulative probability distribution, estimated from local time series. With reference to the whole city, we calculated the total storable volume *V*_*tank*_ as the sum of single contributions. Supposing that 10% of the tank volume can be used each day for domestic non-potable purposes, the daily *Q*_*RWH*_ discharge in case of RWH system installation at large-scale can be estimated from the conservation mass law as:

$$Q_{RWH}=Q_{0}-\frac{V_{i}}{1day}.$$

*V*_*i*_ represents the volume stored in all the water tanks within the city during the *i*-th day, and can be estimated as:

$$V_{i}=\left\{ \begin{array}{ccc} 0 & if & V_{i}^{*}<0\\ V_{i}^{*} & if & 0<V_{i}^{*}<V_{tank}\\ V_{tank} & if & V_{i}^{*}>V_{tank} \end{array}\right.$$

where $V_{i}^{*}={R_{i}*A_{slop}*1d+V}_{i-1}-0.1*V_{tank},$ *R*_*i*_ is the daily rainfall rate at the *i*-th day and *A*_*slop*_ indicates the horizontal projection of the surface of the sloped roofs, where RWH systems are assumed to be installed.

For scenarios (e) and (f), where RWH systems are installed on sloped roofs and GR over flat ones, the runoff discharge can be estimated as:

$$Q_{RWH+GR}=Q_{0}-\frac{V_{i}}{1day}-q_{GR}.$$

### Time between rainfall and runoff peaks, *T*_*lag*_

As presented in the introduction, blue-green solutions are powerful tools, which enable the pluvial flood risk to be mitigated, retaining a fraction of the rainfall and delaying the runoff generation. In order to investigate the latter aspect, we complement our analysis with the computation of an index, *T*_*lag*_, defined as the time required to trigger runoff generation from a blue-green solution, after the beginning of a rainfall event. This index, calculated for each day, is a proxy of lag time between rainfall and runoff in a point that is close to the blue-green considered structure.

Rainfall data available for this study presents a daily temporal resolution, which is generally too coarse to estimate properly the hydrological response time in urban areas [45–47]. For this reason, we investigated different scenarios, where we assumed that the daily rainfall depth is uniformly distributed in a fixed duration τ, equal to 1, 3, 6, 12, 18 and 24 h. When τ is 1 h, the rain volume is assumed to fall in the first hour of the day, while, for τ equal to 24 h, the rainfall event is supposed to be uniform during the entire day. Through this approach, 6 rainfall scenarios with different durations are defined and used to estimate the time after which the runoff is generated from the roofs.

For the investigated blue-green solutions, the runoff generation starts when the soil moisture *s* reaches the value triggering leakage, namely *S*_*fc*_ in the case of GRs, and when the water volume in the tank *V*_*i*_ becomes greater than the total water tank volume *V*_*tank*_ for RWH systems. For each day *i* it is, hence, possible to estimate the volume per surface unit *h*_*lag*_ that must be filled before runoff generation will start:

$${h_{lag}}_{i}=\left\{ \begin{array}{cc} (S_{fc}-s_{i-1})nZ_{r} & for\;GRs\\ \frac{V_{tank}-V_{i}}{A_{slop}} & for\;RWH \end{array}\right.$$

The index *T*_*lag*_ is obtained by dividing the *h*_*lag*_ by the rainfall intensity *R*_*τ*_, calculated as the ratio between the daily rainfall depth and the selected duration τ:

$$T_{lag}=\frac{h_{lag}}{R_{\tau }}$$

Obviously, when *T*_*lag*_ > τ there will not be runoff from the green roof or RWH system, because the system is able to store the entire rainfall volume. In the opposite case, when *T*_*lag*_ < τ, the runoff starts during the rainfall event. In order to have one representative value for each location, the average $\bar{T_{lag}}$ is defined as the mean of the daily *T*_*lag*_ over the entire time series, excluding the days with no rainfall and no runoff.

## Results

### Roof distribution

Roof slope distribution presents high variability from city to city, as shown in Fig 2L1–2L9, where buildings with flat roofs are coloured green, while sloped ones are coloured blue. Small and sloped roofs mainly correspond to private houses, while large and flat roofs usually cover public buildings. It is also evident that the largest fraction of the selected urban areas is covered by sloped roofs in most of the considered cities, except for Airdrie and Montreal (L2 and L4), where flat and sloped areas are approximately equally distributed: here, private houses frequently have flat roofs. Average roof slope of the buildings is a peculiarity of the cultural and architectural background of each study case: it drives the choice of the blue-green solution to be installed and, consequently, the costs and the level of flood mitigation that can be achieved.

### Average lag time between rainfall and runoff

**Fig 3:**
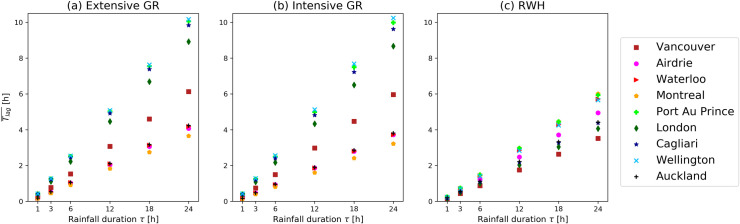
Average lag time between rainfall and runoff $\bar{T_{lag}}$. Six potential rainfall durations corresponding to 1, 3, 6, 12,18 and 24 h have been investigated and plotted for extensive (a) and intensive (b) GRs and for RWH (c) tanks. Different symbols and colours represent the nine selected locations.

The average time between rainfall and runoff $\bar{T_{lag}}$, as defined in the Methodology section, is plotted for each location and for the investigated rainfall durations in Fig 3. $\bar{T_{lag}}$ has been estimated for extensive and intensive GR and for RWH. Each location is highlighted with a different colour and symbol. $\bar{T_{lag}}$ increases for higher rainfall durations. Intensive and extensive GR present similar $\bar{T_{lag}}$, which is close to zero for rainfall events with rainfall duration τ equal to 1 h and varies between 3.2 h and 10.3 h for τ equal to 24 h.

The high variability among the nine selected locations depends on the climatological conditions: cities with long rainy periods, such as Montreal and Waterloo, have a higher probability to have soil moisture close to the leakage triggering point than other cities, such as Cagliari, Wellington or Port Au Prince, which are more likely to have intense rainfall events after long dry periods.

RWH presents $\bar{T_{lag}}$ values generally lower than for GRs: for rainfall events with a duration of 24 h, $\bar{T_{lag}}$ varies between 3.5 h and 6 h. This is due to the fact that the available storing volume for the roof surface unit is higher for GRs than for RWH tanks. The $\bar{T_{lag}}$ variability among cities is lower for RWH systems than for GRs because the maximum storage volume available for GRs is fixed for every location, while the RWH tank volume varies depending on the rainfall characteristic of the city.

The analysis of the average time of peak delay $\bar{T_{lag}}$ shows how GRs enable a higher performance to be achieved in terms of runoff generation delay per roof surface unit, making a better contribution to the mitigation of pluvial flood.

### Discharge reduction from GRs and RWH systems under extreme rainfall events

GR installation cost per unit area depends on the typology: extensive configurations, which are characterized by a thin soil layer and higher flexibility, are less expensive than intensive solutions, which, on the other hand, guarantee a higher retention capacity [12, 48–50]. RWH system efficiency depends on the volume of installed tanks, chosen to be capable of collecting the water that falls over each roof during the extreme rainfall event. Their cost, which can be derived from commercial catalogues [51], depends on the selected volume.

Total costs for the installation over the entire study area of either GRs on flat roofs or RWH systems on sloped ones are detailed in Table 1 as a function of potential outflow reduction percentage Δ*Q*. Costs are referred to the installation over the entire city boundaries and are strongly influenced by the city dimension. GRs and RWH costs include maintenance costs, estimated for the lifetime (50 years) and discounted at the time of construction. Potential additional costs, due to roof reinforcement structures, are not evaluated in this analysis.

**Table 1:** Cost-efficiency summary for each location. Maximum discharge reduction (as a percentage of initial runoff), total cost of the installation of the different solutions in the entire city and effectiveness (ratio between total costs and maximum discharge reduction).

Location	ΔQmax [%]			Total Cost [M€]			Effectiveness E [M€/%ΔQ]		
	GR_ex_	GR_int_	RWH	GR_ex_	GR_int_	RWH	GR_ex_	GR_int_	RWH
**Vancouver (L1)**	0.77	1.00	16.34	519	1299	45	674.03	1299.00	2.75
**Airdrie (L2)**	6.45	7.47	7.71	208	521	1	32.25	69.75	0.13
**Waterloo (L3)**	1.55	1.95	12.22	254	635	16	163.87	325.64	1.31
**Montreal (L4)**	5.04	6.41	7.93	7832	19582	96	1553.97	3054.91	12.11
**Port Au Prince (L5)**	1.51	2.00	36.68	4	10	0.7	2.65	5.00	0.02
**London (L6)**	0.71	0.87	16.9	2293	5733	375	3229.58	6589.66	22.19
**Cagliari (L7)**	1.84	2.21	18.10	86	215	5	46.74	97.38	0.28
**Wellington (L8)**	0.55	0.70	18.14	82	205	20	149.09	293.67	1.10
**Auckland (L9)**	0.77	0.98	13.33	1685	4214	210	2188.31	4300.00	15.75

Table 1 highlights the maximum potential relative discharge reduction Δ*Q*, as a percentage of the unaltered runoff, and the total cost (in M€) required for the realization of 3 investigated scenarios (see Fig 1A, 1B and 1C). Moreover, the effectiveness *E* is also introduced, computed as the ratio between total cost and relative discharge reduction. *E* represents an average estimation of the millions of euros needed to reduce the discharge by 1% compared to the unaltered conditions:

$$E=\frac{Total\;costs\;[M€]}{\Delta Qmax\;[\%]}$$

Lower *E* values correspond to more effective solutions to reduce the discharge. Focusing on GR results, the thicker soil layer of intensive GRs guarantees a larger discharge reduction than extensive ones. However, the best technical performances are not balanced by the costs: to achieve the same Δ*Q*, intensive GRs require, for most of the locations, a financial investment two times larger than for extensive structures: the effectiveness of intensive GRs is generally almost double that of extensive GRs. In the case of Port Au Prince, for example, the intensive GR effectiveness is equal to 5 M€/%Δ*Q*, while the extensive one is 2.65 M€/%Δ*Q*.

On the other hand, RWH systems show to be more efficient and less expensive than GRs, for all the investigated cities, and RWH effectiveness is always lower than that of GRs. Airdrie (L2) and Montreal (L4), however, are characterized by similar values of Δ*Q* for GRs and RWH systems: since the number of flat roofs in these two cities is higher, GR installation allows higher values of Δ*Q* to be obtained compared to the other case studies. In L3 and L5, the maximum outflow reduction is similar for all scenarios, but the installation of RWH tanks over the entire city is still less expensive than the GR installation.

In summary, the comparison between three possible scenarios (Fig 1A–1C) highlights how RWH systems are more cost-efficient than both extensive and intensive GRs, with a higher reduction of the total city outflow at lower costs. RWH effectiveness varies between 0.02 and 22.19, while for intensive GRs between 5 and 6589 M€/%Δ*Q*.

### Long-term simulation of different scenarios

**Fig 4:**
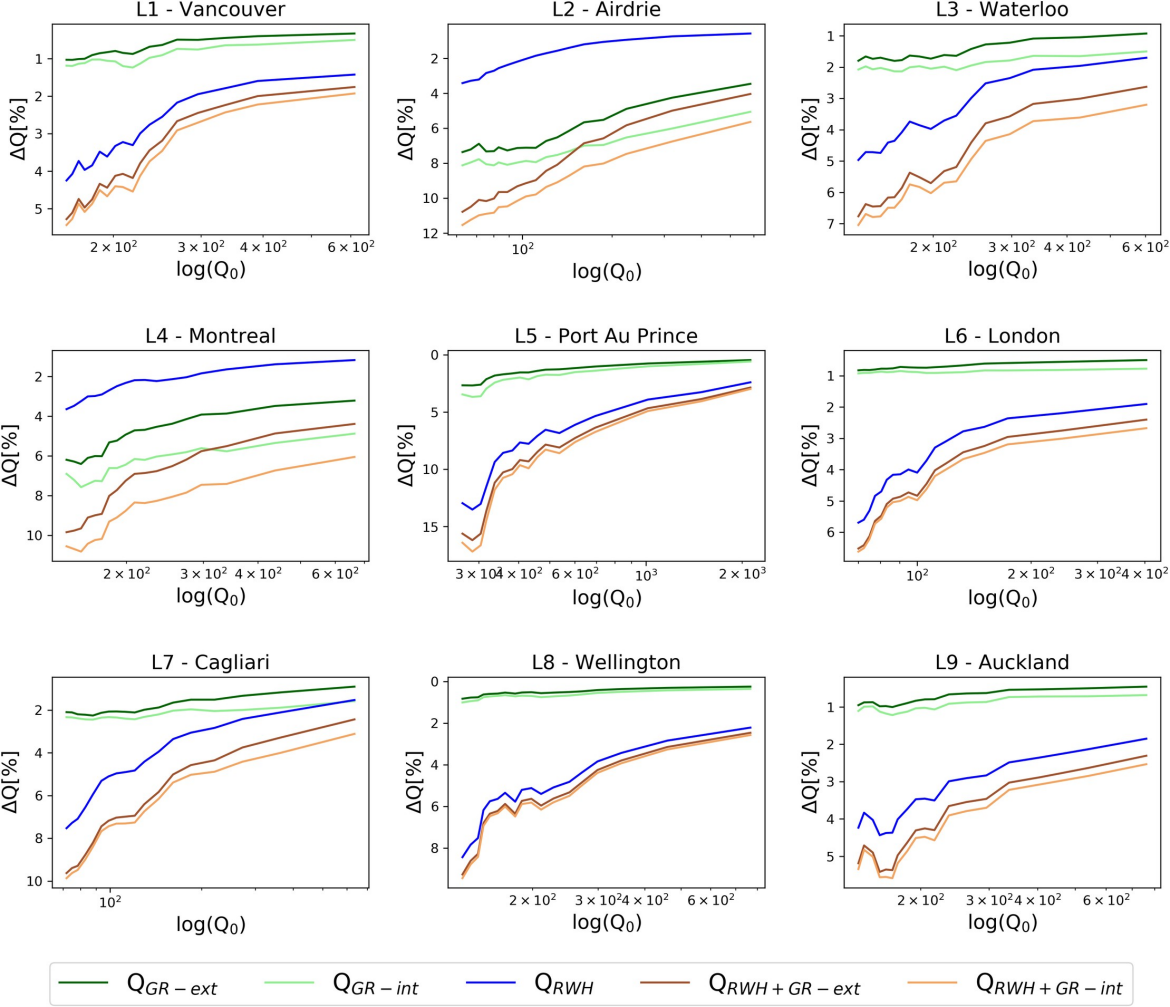
Outflow reduction ΔQ at daily scale as a function of the discharge in unaltered conditions. Moving average outflow reduction ΔQ at daily scale is plotted as a function of the discharge in unaltered conditions at logarithmic scale, for different scenarios in the selected 9 locations. Only events with discharge in unaltered conditions above the 95% quantile are plotted.

The performance of the blue-green mitigation solution is strongly influenced by the antecedent rainfall condition: if the soil is already wet, the retention capacity of GRs is reduced and, similarly, if the tank is partially filled, discharge reduction is consequently affected. To include these physical insights into a dynamic representation, the discharge reduction is investigated at daily scale using a continuous local rainfall time series as input for the models. For each location, Fig 4 plots the average discharge reduction Δ*Q* for each scenario as a function of the discharge *Q*_0_ under unaltered conditions. Five different scenarios are here considered: installation of only extensive (a) or intensive (b) GRs, installation of only RWH systems (c), as in the previous case, and in addition a combination of RWH systems with extensive (d) or intensive (e) GRs. To analyse the most critical outflows corresponding to extreme events that can lead to urban floods, only days with outflow in unaltered conditions *Q*_0_ above the 95% quantile are shown in Fig 4.

As expected, the combination of RWH and intensive GRs always ensures the maximum flow reduction, which is generally higher than 2% in every city for the most extreme events. RWH is generally more efficient than GRs, ensuring a higher outflow reduction. However, for L2 and L4 the large-scale installation of GRs doubles the contribution to the flood reduction with respect to the RWH contribution, thanks to the high percentage of flat roofs in the city (Fig 2, 2L2 and 2L4).

As *Q*_0_ increases (corresponding to more intense rainfall events), we observe a general worsening of the potential efficiency of blue-green solutions, being almost negligible for very intense extremes. In the city of London (L6), for example, the large-scale installation of RWH systems could reduce the runoff from 2% (very extreme rainfall events) up to 6% (extreme events). The runoff generation could be reduced combining RWH on sloped roofs and intensive GRs on flat ones: in this case the reduction can vary between 3% and 7%. The installation of only GRs on flat roofs enables a discharge reduction of 2% in most of the cities to be reached, highlighting the need to combine this solution with RWH systems. Only Airdrie and Montreal present a higher performance, thanks to the high flat roof percentage. For the nine investigated locations, the maximum outflow reduction achievable with a RWH and intensive GR coupled system varies between 5.5% (Vancouver and Auckland) and 17.5% (Port Au Prince).

## Conclusions

The present study highlights and quantifies how the combination of RWH systems and GRs performs in terms of runoff reduction in urban areas. Although the study was conducted under some simplified hypotheses, such as neglecting possible additional costs for structural reinforcement in old buildings before the installation of GRs, it provides an overall comparison on performances of different blue-green solutions worldwide.

Roof distribution within a city exerts crucial constraints in the choice among possible solutions, and thus influences potential flood mitigation performances. Consequently, slope roof distribution analysis can provide a valuable support in deciding the optimal combination of GRs or RWH. For instance, in cities with a prevalence of large flat roof surfaces, discharge reduction is achievable by installing only GRs. In most of the cities, however, good flood risk reduction can be achieved by coupling GRs with RWH systems.

The attenuation of rainfall peak and average time between rainfall and runoff peaks in a city are function of local climate and roof distribution and depends on the kind and extension of the undertaken mitigation measure. GRs are more suitable than RWH in delaying the time between rainfall and runoff peaks, giving a potential higher contribution to the pluvial flood mitigation. For extreme rainfall events with duration of 24 h, the installation of GRs can delay the runoff generation up to 10.3 h, highlighting the high potential of the installation of both extensive and intensive GRs in the urban environment.

In all the investigated locations, the 95% daily rainfall peak reduction varies approximately between 5% and 15%, if both GR and RWH solutions are implemented. The highest benefits arise from RWH systems serving all the sloped roofs, coupled with extensive GRs installed on all the available flat roofs.

Although the quantitative analysis revealed that GR installation is less cost-efficient than RWH in terms of runoff reduction, the additional benefits of GRs should be considered in the general development of the city. Policy makers must account for the thermal insulation benefits, the increase of green areas and biodiversity, the potential carbon sequestration and the added aesthetic value. RWH, on the other hand, may help in flood management at urban scale with relatively small investments. Moreover, the water collected from RWH tanks can be reused for domestic non-potable purposes, contributing to reducing the pressure on the supply systems during dry periods. However, tanks can be difficult to hide and integrate in the urban environment, since they require large available spaces, which can be a limiting constraint for RWH installation in some cities, especially in historical centres. On the other hand, the installation of GRs on old buildings in the city centres might also require additional structures and planning to ensure roof stability. All these factors need to be evaluated to choose the best solution, in order to mitigate pluvial flood in an efficient way and, at the same time, obtain multiple benefits at a lower cost.

The approach presented here is scalable and can be applied to the whole city scale (as in our case), and to small neighbourhoods or to focus only on areas prone to urban floods. For example, this analysis could be carried out for the urban expansion zones: the installation of blue-green solutions on new building will be easier, less expensive and will reduce the impact of the increasing urbanization. Moreover, this approach could be developed using a fully distributed model with spatially and temporally distributed rainfall data. With this approach, it would be possible to include also the position of the blue-green solution installation and to evaluate how this could contribute to the runoff generation reduction. The proposed method can be a powerful tool to support urban planning in reducing and preventing flood risk.

## Supporting information

S1 Table Summary of the data sources for each location. Website of geoportals used to derive DSM, Building shape files and satellite maps. Weather data were derived from the Global Historical Climate Network (GHCN): for each location, the code of the investigated station is reported.
(DOCX)
