# Supplementary material for: Comparison of blue-green solutions for urban flood mitigation: A multi-city large-scale analysis
Source: PLoS One. 2021 Jan 29;16(1):e0246429. doi: 10.1371/journal.pone.0246429 (PMC7845955; doi:10.1371/journal.pone.0246429)
Supplement: S1 Table — Website of geoportals used to derive DSM, Building shape files and satellite maps. Weather data were derived from the Global Historical Climate Network (GHCN): for each location, the code of the investigated station is reported. (DOCX) [file pone.0246429.s001.docx]

**S1 Table Summary of the data sources for each location.** Website of geoportals used to derive DSM, Building shape files and satellite maps. Weather data were derived from the Global Historical Climate Network (GHCN): for each location, the code of the investigated station is reported.

| ID | City | DSM | Buildings and green areas shape files | Weather Station Code |
| --- | --- | --- | --- | --- |
| L1 | Vancouver | <http://ftp.maps.canada.ca/pub/elevation/dem_mne/highresolution_hauteresolution/dsm_mns/1m/> | <https://data.vancouver.ca/datacatalogue/> | CA001108395 |
| L2 | Airdrie | <http://ftp.maps.canada.ca/pub/elevation/dem_mne/highresolution_hauteresolution/dsm_mns/1m/> | <http://data-airdrie.opendata.arcgis.com/> | CA003031093 |
| L3 | Waterloo | <http://ftp.maps.canada.ca/pub/elevation/dem_mne/highresolution_hauteresolution/dsm_mns/1m/> | <http://data.waterloo.ca/> | USC00306047 |
| L4 | Montreal | <http://ftp.maps.canada.ca/pub/elevation/dem_mne/highresolution_hauteresolution/dsm_mns/1m/> | <http://donnees.ville.montreal.qc.ca/dataset> | CA007025250 |
| L5 | Port Au Prince | <http://opentopo.sdsc.edu/datasets?minX=-74.2499957084656&minY=16.299044835995616&maxX=-71.01562714576725&maxY=20.96143360280668> | <https://data.world/hot/65a41adf-1068-4964-be4c-08f435e5d302> | DRM00078482 |
| L6 | London | <https://data.gov.uk/dataset/fba12e80-519f-4be2-806f-41be9e26ab96/lidar-composite-dsm-2m> | <https://data.gov.uk/> | UKE00105915 |
| L7 | Cagliari | <http://www.sardegnageoportale.it/> | <http://www.sardegnageoportale.it/> | IT421300 |
| L8 | Wellington | <https://data.linz.govt.nz/> | <https://data.linz.govt.nz/> | NZM00093110 |
| L9 | Auckland | <https://data.linz.govt.nz/> | <https://data.linz.govt.nz/> | NZ000093417 |
